# BJ-3105, a 6-Alkoxypyridin-3-ol Analog, Impairs T Cell Differentiation and Prevents Experimental Autoimmune Encephalomyelitis Disease Progression

**DOI:** 10.1371/journal.pone.0168942

**Published:** 2017-01-17

**Authors:** Maheshwor Timilshina, Youra Kang, Ishmit Dahal, Zhiwei You, Tae-gyu Nam, Keuk-Jun Kim, Byeong-Seon Jeong, Jae-Hoon Chang

**Affiliations:** 1 College of Pharmacy, Yeungnam University, Gyeongsan, Republic of Korea; 2 Department of Pharmacy and Institute of Pharmaceutical Science and Technology, Hanyang University, Ansan, Republic of Korea; 3 Department of Biomedical Laboratory Science, Daekyeung College, Gyeongsan, Republic of Korea; Wayne State University, UNITED STATES

## Abstract

CD4^+^ T cells are essential in inflammation and autoimmune diseases. Interferon-γ (IFN-γ) secreting T helper (Th1) and IL-17 secreting T helper (Th17) cells are critical for several autoimmune diseases. To assess the inhibitory effect of a given compound on autoimmune disease, we screened many compounds with an *in vitro* Th differentiation assay. BJ-3105, a 6-alkoxypyridin-3-ol analog, inhibited IFN-γ and IL-17 production from polyclonal CD4^+^ T cells and ovalbumin (OVA)-specific CD4^+^ T cells which were activated by T cell receptor (TCR) engagement. BJ-3105 ameliorated the experimental autoimmune encephalomyelitis (EAE) model by reducing Th1 and Th17 generation. Notably, Th cell differentiation was significantly suppressed by BJ-3105 treatment without inhibiting *in vitro* proliferation of T cells or inducing programmed cell death. Mechanistically, BJ-3105 inhibited the phosphorylation of JAK and its downstream signal transducer and activator of transcription (STAT) that is critical for Th differentiation. These results demonstrated that BJ-3105 inhibits the phosphorylation of STAT in response to cytokine signals and subsequently suppressed the differentiation of Th cell responses.

## Introduction

CD4^+^ T cells are pivotal in mediating adaptive immunity. The major function of adaptive immunity is to mount a specific response to a pathogen while minimizing self-reactivity [1]. Naïve T cells differentiate into effector cells with functional potential for orchestrating pathogen clearance under the guidance of cytokines produced by innate immune cells [2]. Differentiation of the naïve CD4^+^ T cells require antigenic stimulation through T cell receptor (TCR) and CD4 as a co-receptor with major histocompatibility complex class-II (MHC-II) molecule presented by antigen presenting cells [3]. During the TCR activation and antigenic stimulation in the presence of specific cytokine milieu, naïve CD4^+^ T cells differentiate into Th1, Th2, Th9, Th17 and T regulatory cell (Treg). Each T cell lineage produces its own set of cytokines [1, 4]. Th1 effector cells regulate immunity against infectious intracellular pathogens [1]. Th17 cells are specialized to enhance immunity against extracellular bacterial infections by recruiting neutrophils [2, 5]. However, excessive activation of Th1 and Th17 cells is important in chronic inflammation and involved in immunopathology of autoimmune diseases [1, 6].

Upon antigenic stimulation, naïve CD4^+^ T cells proliferate and differentiate into Th1 and Th17 effector subsets associated with production of proinflammatory cytokines [7]. Development of Th1 cells from naïve CD4^+^ T cells is driven by activation in the presence of IL-12 cytokine and by induction of Th1 specific transcription factor T-bet [8]. Th17 cells develop from naïve CD4^+^ T cells driven by activation in the presence of TGF-β and IL-6 induced by Th17 specific transcription factor retinoic acid-related orphan receptor γ t (RORγt). Different transcription factors direct the differentiation of different T cell lineages based on the cytokine milieu. RORγt directs interleukin-17 (IL-17) secreting T cells [9–11]. T-bet, GATA-3 and Foxp3 direct Th1, Th2 and T regulatory cells, respectively [12, 13]. Although the role of each subset in autoimmunity is open to debate, it is widely accepted that Th1 and Th17 cells are proinflammatory and responsible for inducing autoimmunity and inflammatory processes.

Autoimmunity is the dysregulated pathological immune response that attacks self-tissues. Multiple sclerosis (MS) is an autoimmune disease characterized by inflammation of the central nervous system, destruction of the myelin sheath around the axon of neurons and finally paralysis mediated by myelin specific CD4^+^ T cells [14]. EAE is an established model of MS, with clinical and histopathological preclinical similarities to human MS disease [15, 16]. Although the specific causes of MS are not clear, genetic background and environmental factors may contribute to the etiology [17–20]. MS has auto-reactive T cell migration and infiltration into the central nervous system (CNS) and subsequent demyelination of the axon in the brain and spinal cord [17, 18]. Differentiated Th1 and Th17 cells migrate across the blood-brain barrier, and initiate and maintain MS in human and EAE in mice [9, 21]. A number of drugs are approved to treat MS, although there are serious side effects and response failures [22–24]. There is an unmet need for safe medications to manage autoimmune and inflammatory diseases. We investigated a novel compound that can reduce inflammation by inhibiting the differentiation of naïve CD4^+^ T cell to Th1 and Th17 cells.

Complex network of cytokines and transcription factors regulate the differentiation and function of Th1 and Th17 cells. The JAK/STAT pathway is a signaling pathway used by numerous cytokines that also regulates differentiation and function of Th1 and Th17 cells [12, 25, 26]. Intracellular signaling pathway such as Janus Kinas (JAK) pathway have emerged as key hubs in the cytokine network [27]. Differentiation of naïve CD4^+^ T cells into Th lineages involves up-regulation of signal transducer and activator of transcription (STAT) proteins [28]. Activation of STAT1 and STAT4 is important for Th1 differentiation whereas STAT3 activation is crucial for Th17 differentiation [1, 29]. Although STAT is important in T cell development, differentiation and immune defense, unrestrained activation of STATs has pathological implications for autoimmunity [30]. STATs are an attractive pharmacological target for treatment of Th1 and Th17 cell mediated immune disorder because they are central in T cell function [1, 30, 31].

While screening compounds, we found that a 6-alkoxypyridin-3-ol analog, BJ-3105 (2,4,5-trimethyl-6-(3-phenylpropoxy)pyridin-3-ol) significantly suppressed Th cell function by inhibiting Th1 and Th17 differentiation and marginally decreasing the proliferation of the activated T cells without apoptotic effect. These findings prompted us to investigate BJ-3105 as a treatment for autoimmune diseases like MS. We found that BJ-3105 treatment prevented the onset of EAE and alleviates ongoing EAE by reducing Th1 and Th17 cells in the spleen, draining lymph nodes and CNS of EAE inflicted mice. We further investigated the molecular mechanism underlying the BJ-3105 mediated effect and found that BJ-3105 attenuated differentiation of naïve CD4^+^ T cells by inhibiting phosphorylation of JAK1 and JAK2 and its downstream STAT1 and STAT4 in Th1 cell and phosphorylation of STAT3 in Th17 cell. This study therefore revealed a novel immune regulatory function of BJ-3105 by inhibiting Th1 and Th17 by modulating the JAK-STAT pathway.

## Materials and Methods

### Mice

C57BL/6 mice and OT-II mice were purchased from Jackson Laboratory and housed under specific pathogen–free conditions and animal health were monitored every day in the animal care facility at the Yeungnam University animal care center. Experiments were carried out according to the institutional Guide for Care and Use of Laboratory Animals. CO_2_ inhalation using gradual fill method was used as method of euthanasia to minimize potential pain. No animals were died during the study. The experiment protocol was reviewed and approved by Institutional Animal Care and Use Committee at Yeungnam University.

### Flow cytometric analysis

For intracellular cytokine staining, cells were re-stimulated in complete medium with phorbol 12-myristate 13-acetate (PMA) (50 ng/ml) (Sigma) and ionomycin (750 ng/mL) (Calbiochem) with protein transport inhibitors, GolgiStop (BD Biosciences) in a cell incubator with 5% CO_2_ at 37°C for 4 h. Cell surface staining was done in 1× PBS with FITC-conjugated anti-CD4 (GK1.5; Biolegend), PE-Cyanine7-conjugated anti-B220 (RA3-6B2; Biolegend), APC-conjugated anti-CD3ε (145-2C11 Biolegned), PE-Cyanine7-conjugated anti-CD8a (53–6.7;Biolegend), PE-conjugated CXCR3 (CXCR3-173 Biolegend), PE-conjugated CCR6 (29-2L17 Biolegend). After surface markers staining, cells were fixed and permeabilized using fixation and permeabilization buffer (BD bioscience) according to manufacturer’s instructions. For Foxp3 staining cells were fixed and permeabilized using fixation and permeabilization buffer (ebioscience) according to manufacturer’s instructions. Cells were stained with fluorescence-conjugated cytokine antibodies using PE-conjugated anti-IFN-γ (XMG;Biolegend), APC-conjugated anti-IL-17-A (TC11-18H10.1;Biolegend), PE-conjugated anti-CD25 (PC61;Biolegend), APC conjugated anti-Foxp3 (FJK-16s;eBioscience), at 4°C or ice for 15 min before analysis. Antibodies against phospho-JAK1 (Y1022/1023;Cell signaling), p-JAK2 (Y1007/1008;Cell signaling), phospho-STAT1 (Tyr^701^), phopho-STAT3 (Tyr^705^) and phospho-STAT4 (Tyr^693^) used for immunoblot and flow cytometry were from BD Biosciences. Cells were analyzed with BD FACS Verse flow cytometer (BD Biosciences), and data were analyzed with FlowJo software.

### *In vitro* T cell differentiation assay

Naïve CD4^+^ T cells from spleen and lymph nodes of the 6–8 weeks C57BL/6 mice were sorted by positive selection with CD4-conjugated magnetic beads (MiltenyiBiotec). CD4^+^ T cells (2×10^5^/well) were activated by plate bound anti-CD3 (5 μg/ml) (145-2C11; Biolegend) and Anti-CD28 (1 μg/mL) (37.51; eBioscience) in the presence of tofacitinib or BJ-3105 for 72 h under cell culturing conditions (37°C, 5% CO_2_). For antigen specific Th cell differentiation, OVA-specific naïve CD4^+^ T cells from spleen and lymph nodes of OT-II mice were isolated and sorted by positive selection with CD4-conjugated magnetic beads (MiltenyiBiotec). CD4^+^ T cells (2×10^5^/well) and irradiated APCs (1×10^5^/well) were co-cultured in the presence of OVA_323–339_ (0.1 μM) with tofacitinib or BJ-3105 for 72 h under cell-culturing conditions (37°C, 5% CO_2_). Th cell differentiation conditions were as follows: for Th1 differentiation, IL-12 (10 ng/mL) (Biolegend) plus anti–IL-4 (5 μg/mL) (Biolegend); for Th2 differentiation, IL-2 (20 ng/ml) (Biolegend), IL-4 (100 ng/ml) (Biolegend) plus anti–IFN-γ (5 μg/mL) (Biolegend) and anti-IL-12/23) (Biolegend); for Th9 differentiation, IL-2 (20 ng/ml) (Biolegend), TGF-β1 (10 ng/mL) (R&D system), IL-4 (10 ng/ml) (Biolegend) plus anti–IFN-γ (5 μg/mL) (Biolegend); for Th17 differentiation, TGF-β1 (1 ng/mL) (R&D system), IL-6 (10 ng/mL) (R&D system) plus anti-IL-4 (5 μg/mL) (Biolegend) and anti–IFN-γ (5 μg/mL) (Biolegend); for Treg differentiation, IL-2 (10 ng/ml) (Biolegend), TGF-β1 (10 ng/mL) (R&D system) plus anti-IL-4 (5 μg/mL) (Biolegend) and anti–IFN-γ (5 μg/mL) (Biolegend) and culture in a cell incubator with 5% CO_2_ at 37°C for 72 h.

### T cell proliferation assay

For the proliferation assay, naïve CD4^+^ T cells were purified by MACS beads (MiltenyiBiotec) and were labeled with CFSE (eBioscience) at 37°C water bath for 15 min. Labelled cells were stimulated in 96 well culture plates with plate bound anti-CD3 (5 μg/mL) and anti-CD-28 (1 μg/mL) in the presence of DMSO or BJ-3105 in Th1 and Th17 differentiation conditions: Th0 (anti-CD3/CD28 with no cytokines), Th1 (IL-12, 10 ng/mL plus anti-IL-4, 5 μg/mL), Th17 (IL-6, 10 ng/mL, TGF-β, 1 ng/ml plus anti-INF-γ and anti-IL-4, each at 5 μg/mL). After 72 h of culture, cell proliferation was assessed by CFSE dilution, using flow cytometry. For 5-bromo-2'-deoxyuridine (BrdU) labeling, naive CD4^+^ T cells from spleens and lymph nodes were isolated by immunomagnetic positive selection from 6–10 weeks C57BL/6 mice. The cells were cultured under Th1 differentiation condition with BrdU (10 μM). After 72 h of culture, cells were stained with APC-conjugated BrdU as per manufacturer’s protocols (BD Biosciences) in BrdU kit and cell proliferation was assessed by using flow cytometry. For Ki-67 detection, naive CD4^+^ T cells were isolated and cultured under Th1 differentiation condition and after 72 h of culture; cells were stained with PE-conjugated Ki-67 (Biolegend). Cell proliferation was assessed by using flow cytometry.

### Apoptosis assays

Naive CD4^+^ T cells from spleen and lymph nodes were purified by immunomagnetic positive selection and isolated cells were stimulated in 96 well culture plates with plate bound anti-CD3 (5 μg/mL) and anti-CD-28 (1 μg/mL) in the presence of DMSO or BJ-3105 in Th1 (IL-12, 10 ng/ml plus anti-IL-4, 5 μg/mL) differentiation conditions in a cell incubator with 5% CO_2_ at 37°C for 72 h. Cells were stained with Annexin V-APC and Propidium Iodide as per the manufacturer’s protocols (BD Biosciences) and analyzed by flow cytometry.

### EAE induction, prevention, and treatment

C57BL/6 mice (8–12 weeks old) were immunized subcutaneously with 6 mg/mL synthetic peptide of myelin oligodendrocyte glycoprotein (MOG_35–55_) peptide (MEVGWYRSPFSRVVHLYRNGK, AnaSpec). The immunization was prepared by mixing MOG_35–55_ peptide in CFA containing 10 mg/mL heat-killed H37Ra, strain of *Mycobacterium tuberculosis* (Difco Laboratories). In addition, the animals received 250 ng pertussis toxin (List Biological Laboratories) intraperitoneally (I.P.) on days of immunization and 48 h later. For treatment of EAE, BJ-3105 or DMSO (Sigma-Aldrich) as vehicle control were diluted in 1× PBS and administered at 3 mg/kg on day 0, and continued for every other day. The severity of EAE was monitored and graded on a clinical score of 0 to 5 with 0.5 for intermediate scores: 0 = no clinical signs; 1 = flaccid tail; 2 = paraparesis (weakness, incomplete paralysis of one or two hind limbs); 3 = paraplegia (complete paralysis of two hind limbs); 4 = paraplegia with forelimb weakness or paralysis; 5 = moribund or dead.

### Immunoblotting

Naive CD4^+^ T cells from spleens and lymph nodes were isolated by immunomagnetic positive selection from 6–10 weeks C57BL/6 mice. The cells were cultured in Th1 and Th17 differentiation conditions for 72 h with indicated concentration of compound. Splenocytes and CNS mononuclear cells were obtained from drug treated and untreated EAE mice. Cell pellets were harvested and protein samples were prepared using RIPA buffer containing protease and phosphatase inhibitors. BCA protein assay reagents were used to quantify protein content. Equal amounts of protein were separated by SDS-PAGE and transferred to Polyvinylidene Difluoride (PVDF) membrane for 1 h. Membranes were blocked in 5% bovine serum albumin (BSA) in Tris-buffered saline (TBS)- Tween 20 (TBS-T) for phosphorylated and total protein for 1 h followed by incubation with primary antibodies for overnight at 4°C. The membranes were washed and incubated with secondary antibody in BSA for 1 h at room temperature. The protein bands were detected using ECL kit (Pierce) under a luminescent image analyzer. The membranes were reprobed with β-actin antibody for loading control.

### Cytokine binding assay

Naïve CD4^+^ T cells from spleens and lymph nodes were activated and cultured with plate bound anti-CD3 (5 μg/mL) and anti-CD-28 (1 μg/mL) in Th1, Th2, Th9 and Th17 differentiation conditions for 72 h with indicated dose of BJ-3105. Cell pellets were washed with PBS and rested for 24 h with media containing 10% FBS at 37°C, 5% CO_2_. Cells were stimulated with PMA (50 ng/mL) and ionomycin (750 ng/mL) for 24 h, and harvested proteins were quantified with cytokine binding assay kit (740005; Biolegend) by flow cytometer.

### Histopathology

To assess the degree of CNS inflammation and demyelination, immunized C57BL/6 mice treated with vehicle or BJ-3105 were anesthetized and perfused by intracardiac injection of PBS containing 4% paraformaldehyde. Paraffin embedded 3 μm section of brain and spinal cord were stained with H&E and Luxol fast blue and then examined by light microscopy.

### BrdU incorporation in EAE mice

C57BL/6 mice were treated with 0.8 mg/ml 5-Bromo-2’-deoxyuridine (BrdU) in their drinking water. BrdU solution was prepared in sterile water, protected from light and changed daily. During the continuous labeling phase, EAE induced mice received BrdU up to 15 days. The mice were sacrificed, splenocytes and CNS infiltrated mononuclear cells were analyzed BrdU incorporation using the BrdU Flow Kit (BD Pharmingen) according to the manufacturer’s instructions.

### Generation of bone marrow-derived dendritic cells

Bone marrow cells were flushed from the femurs and tibias of female C57BL/6 mice (6–8 wk) and depleted of red blood cells. Cells were cultured at 2×10^5^ cells per well in 60×15 mm culture discs in medium supplemented with 20 ng/ml recombinant mouse granulocyte macrophage colony stimulation factor (GM-CSF). Nonadherent cells were removed and fresh medium was added every 3 days. On day 8, nonadherent cells released spontaneously from proliferating cell clusters were collected and cultured with BJ-3105 or vehicle under the condition of 200 ng/ml lipopolysaccharide (LPS) stimulation for 4 h.

### Quantitative real time PCR

Total RNA was obtained using Trizol reagent (Invitrogen) and cDNA was synthesized with Goscript Reverse Transcription system (# A5001, Promega Corporation, WI, USA). Level of mRNA expression of each gene was measured with Real-Time PCR system using QuantiTect SYBR Green PCR kit (Qiagen). The following primer pairs were used: *Il12a*, 5’-CCACCC TTGCCCTCCTAAAC–3’ and 5’-GGCAGCTCCCTCTTGTTGTG–3’; *Il27*, 5’-CTCTGCTTCCTCGCTACCAC–3’ and 5’-GGGGCAGCTTCTTTTCTTCT–3’; *Il23*, 5’-AAGTTCTCTCCTCTTCCCTGTCGC–3’ and 5’-TCTTGTGGAGCAGCAGATGTGAG–3’; *Il6*, 5’-TATGAAGTTCCTCTCTGCAAGAGA–3’ and 5’- TAGGGAAGGCCGTGGTT–3’; *Il1b*, 5’-AAGGAGAACCAAGCAACGACAAAA–3’ and 5’-TGGGGAACTCTGCAGACTCAAACT–3’; *Il10*, 5’-ATAACTGCACCCACTTCCCAGTC–3’ and 5’-CCCAAGTAACCCTTAAAGTCCTGC–3’*; β-actin*, 5’-TGTCCACCTTCCAGCAGATGT–3’ and 5’-AGCTCAGTAACAGTCCGCCTAGA.

### Statistical analysis

Data are presented as mean±S.E.M. of three independent experiments. Statistical significance was calculated by Student’s t test or one-way ANOVA (Graph Pad Prism 5.0 software, San Diego, CA, USA). FACS data were analyzed with Flowjo software. Null hypotheses of no difference were rejected if p-values were less than 0.05.

## Results

### BJ-3105 decreases the percentage of Th1 and Th17 cells differentiation

**Fig 1:**
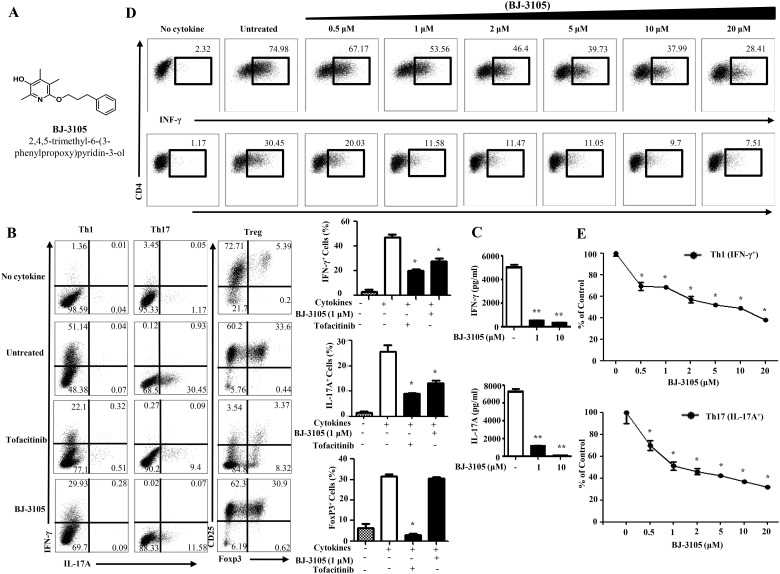
BJ-3105 decreases the percentage of Th1 and Th17 cells differentiation. (A) Chemical Structure of BJ-3105 (2,4,5-trimethyl-6-(3-phenylpropoxy)pyridin-3-ol). (B) Purified naïve mouse CD4^+^ T cells from spleens and draining lymph nodes were stimulated in Th1 and Th17 polarizing conditions for 72 h and Treg for 96 h in the presence of BJ-3105 (1 μM) or tofacitinib (1 μM). CD4^+^ T cells were then restimulated with PMA, ionomycin and golgistop for 4 h and analyzed by intracellular cytokine staining by flow cytometer. The untreated controls were cultured in the presence of DMSO. Percentage of IFN-γ^+^ Th1 cells, IL-17A^+^ Th17 cells and Foxp3^+^ Treg cells were shown in bar diagram. (C) IFN-γ and IL-17A cytokine produced by CD4^+^ T cells were quantified by cytokine binding assay. (D) Th1, Th17 differentiation with different dose of BJ-3105. (E) Compilation of data from three individual experiments showing the inhibitory effect of different dose of BJ-3105 on Th1 (Upper) and Th17 (bottom) cytokine production were shown. For each cytokine, data were normalized to the percentage of cytokine producing cells in the absence of BJ-3105. Representative results of three experiments are shown. *p<0.05, **p<0.001 compared with drug untreated group. Data shown are mean±SEM.

CD4^+^ T cells are essential in the immune response, and Th1 and Th17 cells are most extensively studied for understanding inflammation and autoimmune diseases [32, 33]. Inhibition of IFN-γ producing Th1 and IL-17 producing Th17 cells helps to mitigate autoimmune disease [6]. Exploring the inhibitory effects of candidate compounds on autoimmune disease, we screened with an *in vitro* Th differentiation assay. Our screening revealed potential in a small molecule named BJ-3105 (Fig 1A). To determine whether BJ-3105 influences T cell differentiation, CD4^+^ T cells were purified and cultured under Th1, Th17 and Treg skewing cytokine conditions with or without BJ-3105. We found that BJ-3105 (1 μM) treatment significantly reduced IFN-γ and IL-17 production like commercial tofacitinib at day 3 after *in vitro* stimulation with TCR and cytokine (Fig 1B). In contrast, tofacitinib critically reduced Treg differentiation but BJ-3105 has very less impact on Treg differentiation. The amount of IFN-γ and IL-17 cytokine produced by CD4^+^ T cells were decreased drastically by BJ-3105 treatment (Fig 1C). The amount of IL-4, IL-5 and IL-13 cytokines produced by CD4^+^ T cells were significantly reduced by BJ-3105 treatment in Th2 polarizing condition, but IL-9 cytokine was increased by BJ-3105 in Th9 polarizing condition (S1 Fig). So, the effect of BJ-3105 is different with that of tofacitinib on T cell biology because tofacitinib inhibited all Th generation including Th1, Th2, Th9, Th17 and Treg (Fig 1B and S1 Fig). To further investigate the immune modulatory effects of BJ-3105 on the differentiation of CD4^+^ T cells, varying doses of BJ-3105 were added into TCR and cytokines stimulated CD4^+^ T cells. The levels of IFN-γ and IL-17A were all decreased by this compound in a dose-dependent manner (Fig 1D and 1E). To rule out the possible effect of BJ-3105 on innate myeloid cells, we generated the bone marrow-derived dendritic cells (BMDC) *in vitro*. BMDC was cultured in the presence of BJ-3105 or vehicle under condition of LPS stimulation for 4 hr and found that there was no significant effect of BJ-3105 on mRNA expression of several inflammatory cytokines produced by DCs (S2 Fig). Altogether, we concluded that BJ-3105 significantly and selectively regulate the expression of pro-inflammatory cytokines in CD4^+^ T cells stimulated with TCR and cytokines *in vitro*.

### BJ-3105 decreases the percentage of antigen-specific Th1 and Th17 cell differentiation

**Fig 2:**
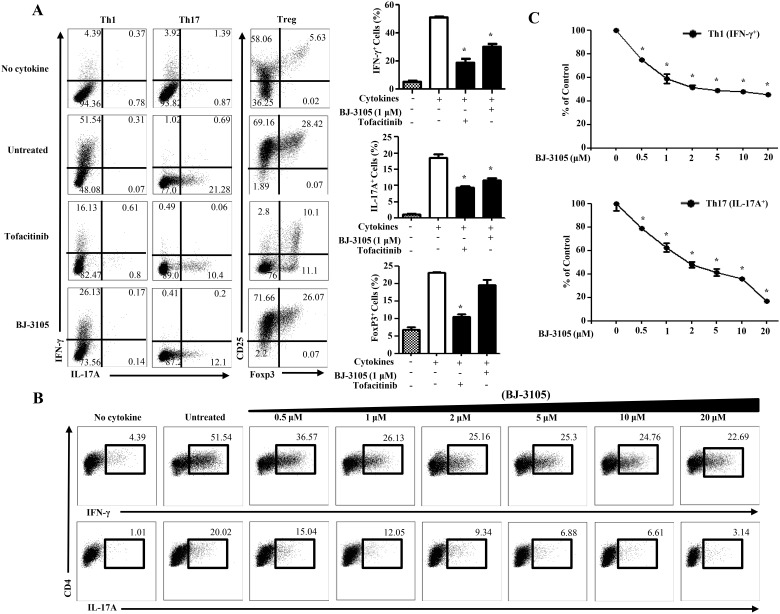
BJ-3105 decreases the percentage of antigen-specific Th1 and Th17 cell differentiation. (A) Naïve CD4^+^ T cells and antigen presenting cells from spleens and lymph nodes were isolated by immunomagnetic positive selection from 6–10 weeks OT-II mice. CD4^+^ T cells and irradiated antigen presenting cells were cultured in Th1, Th17 and Treg cells differentiation conditions in the presence of OVA_323–339_ (0.1 μM) with BJ-3105 (1 μM) or tofacitinib (1 μM). CD4^+^ T cells were then restimulated with PMA, ionomycin and golgistop for 4 h and analyzed by intracellular cytokine staining by flow cytometer. The untreated controls were cultured in the presence of DMSO. (B) The cells were cultured in Th1 and Th17 cells differentiation conditions for 72 h with different concentration of BJ-3105 and analyzed by flow cytometer. (C) Compilation of data from three individual experiments showing the inhibitory effect of different dose of BJ-3105 on Th1 (Upper) and Th17 (bottom) cytokine production were shown. For each cytokine, data were normalized to the percentage of cytokine producing cells in the absence of BJ-3105. Representative results of three experiments are shown. *p<0.05, compared with drug untreated group. Data shown are mean±SEM.

To clarify whether BJ-3105 directly inhibits Th differentiation regardless of antigen type, we used ovalbumin (OVA)-specific OT-II T cell receptor (TCR) transgenic mice, which recognizes OVA_323-339_ in the context of class-II MHC [34]. An *in vitro* culture of naïve OT-II CD4^+^ T cells with tofacitinib and BJ-3105 in the presence of OVA peptide and APCs significantly suppressed the generation of both IFN-γ and IL-17A-secreting cells except Treg generation (Fig 2A). To further characterize the immune modulatory effects of BJ-3105 on the OVA-specific differentiation of CD4^+^ T cells, varying doses of BJ-3105 were added into OVA peptide and cytokines stimulated OT-II CD4^+^ T cells. The levels of IFN-γ and IL-17A were all decreased by this compound in a dose-dependent manner (Fig 2B). It is notable that the BJ-3105-mediated suppression of IL-17A seemed more sensitive than that of IFN-γ because high dose (20 μM) BJ-3105 decreased Th1 by 50%, and decreased Th17 differentiation by more than 80% (Fig 2C). Thus, BJ-3015 compound directly inhibits T cell differentiation without Ag specificity.

### BJ-3105 slightly regulates T cell proliferation without inducing cell death

**Fig 3:**
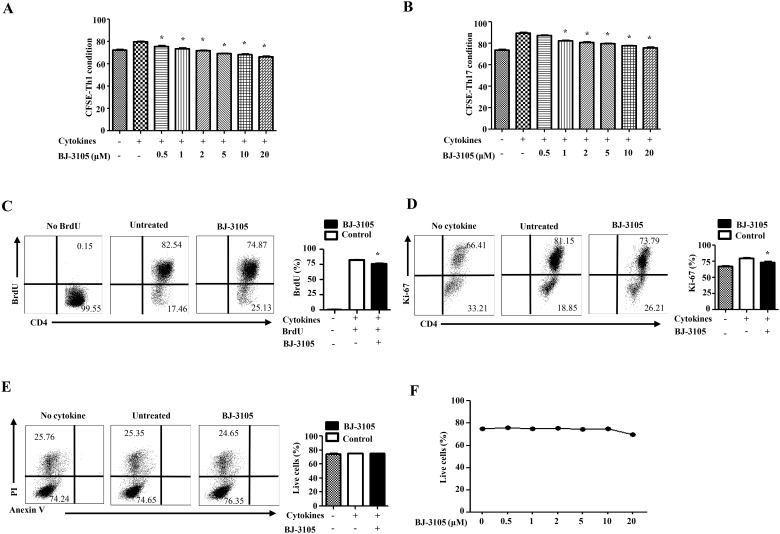
BJ-3105 slightly inhibits CD4^+^ T cell proliferation without inducing apoptosis. CFSE-labeled naïve CD4^+^ T cells were activated with anti-CD3 anti-CD28 under the treatment of indicated dose of BJ-3105 for 3 days in (A) Th1 and (B) Th17 differentiation conditions. Cell proliferation was monitored by assessing CFSE dilution by flow cytometry. (C) Naïve CD4^+^ T cells and APCs from spleens and lymph nodes were isolated and activated under Th1 differentiation condition in the presence of BrdU (10 μM) with or without BJ-3105 (1 μM) and analyzed after 3 days by flow cytometer. Representative bar diagram for percentage of BrdU^+^CD4^+^ T cells were shown. (D) Representative Dot plots examining CD4^+^ T cell proliferation by detecting Ki-67 in Th1 differentiation condition with or without BJ-3105 by flow cytometer. Percentage of Ki-67 positive CD4^+^ T cells were shown. (E) Live cells were detected by Anexin-V and PI staining after cultured in Th1 differentiation conditions for 72 h and analyzed by flow cytometer. (F) Percentage of live cells on dose dependent treatment of BJ-3105. Representative results of three experiments are shown. *p < 0.05, compared with drug untreated group. Data shown are mean ± SEM.

To investigate whether the reduced generation of Th cells by BJ-3105 was caused by a reduced proliferation or increased apoptosis, CFSE-labeled CD4^+^ T cells were cultured with serial doses of BJ-3105 in Th differentiation condition for three days. Based on CFSE dilution, proliferation of IL-12 cytokine treated group was enhanced compared with that of no cytokine-treated group while proliferation of BJ-3105-treated group was slightly decreased dose-dependently (Fig 3A). Similarly, IL-6 and TGF-β increased the proliferation of CD4^+^ T cells with TCR stimulation *in vitro*, but BJ-3105 weakly affected the proliferation of primed T cells by TCR and cytokines (Fig 3B). For analyzing DNA level for proliferation, we used a bromodeoxyuridine (BrdU) cell proliferation assay. Consistently, *in vitro* proliferation by thymidine analog BrdU labeling assay demonstrated that BJ-3105 treatment induced a slight decrease of proliferation under Th1 polarizing condition (Fig 3C). Ki-67, a nuclear protein that is related with cell proliferation, was analyzed after T cell activation with TCR and cytokines. BJ-3105 compound reduced Ki-67 expression rate less than 10% compared with no compound-treated group (Fig 3D). Thus, BJ-3105 may slightly regulate the proliferation of T cells but this effect is less than the inhibitory effect on T cell differentiation by BJ-3105.

Next we investigated whether the apoptosis of primed CD4^+^ T cells causes a reduction in T cell differentiation by BJ-3105. CD4^+^ T cells were cultured with TCR and cytokines in the presence of the BJ-3105 or PBS. On day 3 after activation, apoptosis was assayed by PI and Annexin-V staining. The percentage of viable cells was comparable between PBS and BJ-3105 treated group (Fig 3E). To further explore the effect of BJ-3105 on apoptosis, we cultured CD4^+^ T cells with TCR and cytokines in the presence of serial doses of BJ-3105. Still, there was no significant deleterious effect of high dose compound on *in vitro* T cell activation (Fig 3F). We conclude that although BJ-3105 slightly affects the proliferation of CD4^+^ T cells, the inhibition of T cell differentiation by BJ-3105 is not caused by less proliferation or more apoptosis.

### Treatment of BJ-3105 ameliorates EAE by decrease of inflammatory T cells

**Fig 4:**
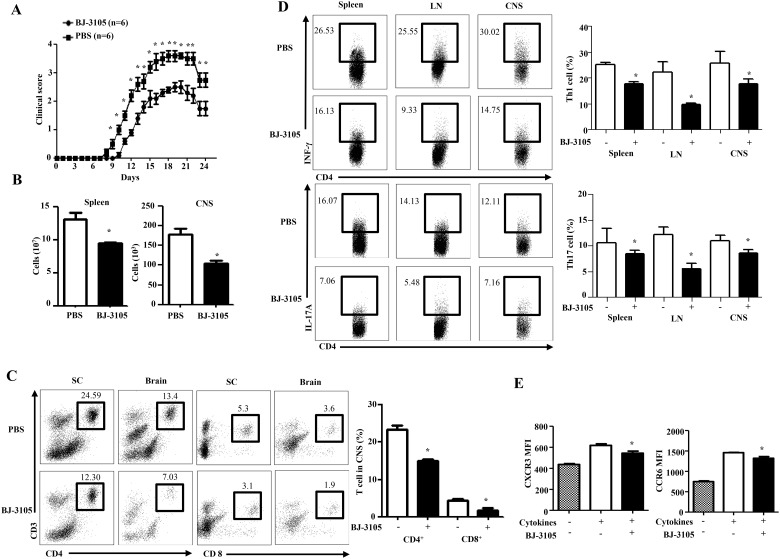
BJ-3105 treatment suppresses development of EAE by decreasing autoreactive Th1 and Th17 generation. Active EAE was elicited in 8–12 weeks female C57BL/6 mice by immunization with MOG_35-55_. The mice were subcutaneously treated with vehicle or BJ-3105 (3 mg/kg/every other day) from day 1 post-immunization as detailed under materials and methods. Mice were monitored for signs and symptoms of EAE. (A) Clinical score were recorded every day. (B) Total cell count in spleen and CNS of drug treated and untreated EAE mice. (C) At 24 days after challenge, total mononuclear cell obtained from the brains and spinal cord of MOG_35–55_ immunized wild-type mice and BJ-3105-treated mice. Total percent of infiltrated CD4^+^ T cells and CD8^+^ T cells in CNS. (D) After 24 days of EAE, lymphocytes were isolated from the spleen, lymph nodes and spinal cord of mice. The fraction of IFN-γ and IL-17A producing CD4^+^ T cells from spleen, draining lymph node and CNS were determined by intracellular staining and analyzed by flow cytometry. Percentage of Th1 and Th17 cells were shown. (E) Flow cytometry analysis of CXCR3 expression on activated T cells under Th1 and CCR6 expression under Th17-polarizing conditions with or without BJ-3105 (1 μM). Bar diagram showing the mean fluorescence intensity (MFI) of CXCR3 and CCR6. Mean±SEM of multiple mice are shown. *p < 0.05, compared with drug untreated group.

The inhibition of Th1 and Th17 cell differentiation by BJ-3105 in *in vitro* prompted us to examine if this compound also affects antigen-specific Th cell responses *in vivo*. To test this, we used the EAE model, because Th1 and Th17 cells are critical for progression and pathology of EAE [35]. Mice were induced for EAE and disease scores were analyzed for the inhibitory effect of BJ-3105. Of note, most BJ-3105-treated mice were resistant to the development of EAE compared with PBS-treated group (Fig 4A). The total cell number from spleen and CNS were also decreased in drug treated EAE mice (Fig 4B). Since autoreactive CD4^+^ T cells, especially Th1 and Th17, are critical to induce EAE, we analyzed Th cells in EAE mice. To this end, mononuclear cells in brain and spinal cord were enriched by density gradient and analyzed by flow cytometry. Significantly fewer infiltrated CD4^+^ and CD8^+^ T cells were present in the brain and spinal cord of the BJ-3105-treated EAE mice than in the same tissue of PBS-treated EAE mice (Fig 4C). Th cells were further analyzed in spleen, draining lymph node (dLN) and central nervous system (CNS) of EAE mice with and without BJ-3105 treatment. As expected, Th1 and Th17 cells were predominant in the spleen and dLN of EAE-induced mice when compared with the untreated wild-type mice (data not shown). Of note, treatment with BJ-3105 significantly reduced the numbers of Th cells in the spleen, dLN and CNS of EAE-induced mice (Fig 4D). The proportion of Th1 and Th17 cells from dLN were strongly decreased in BJ-3105 treated-EAE mice. Histological characterization of the brain and spinal cord revealed less inflammatory infiltrates and less demyelination in the BJ-3105 treated EAE mice (S3A Fig). To find out whether the decrease in Th1 and Th17 cells is due to decrease in proliferation, we use *in vivo* labeling of BrdU through drinking water in BJ-3105 treated and untreated EAE mice. In contrast with previous *in vitro* proliferation data, drug treatment reduced BrdU^+^CD4^+^ T cells in spleen and CNS mononuclear cells (S3B Fig). But we analyzed BrdU^+^CD4^+^ T cells at 14 day after EAE induction, so less proliferation of T cells in BJ-3105 treated EAE mice may be related with less inflammation not by the direct effect of BJ-3105.

Various studies reported that the CXCR3-CXCL11 and CCR6-CCL20 axis plays an essential role in controlling the entry of Th1 and Th17 cells into the CNS and hence mediates the initiation of EAE [19, 36]. In our study we also found significantly reduced percentage of CD4^+^ T cells in to CNS following BJ-3105 treatment (Fig 4C). To investigate the effect of BJ-3105 on the migration of Th1 and Th17 cells to CNS, we analyzed the CXCR3 expression in Th1 and CCR6 expression in Th17 polarizing conditions. The level of CXCR3 expression on Th1-polarizing condition and CCR6 expression on Th17-polarizing condition was slightly down in drug-treated group (Fig 4E) but this inhibitory effect of CXCR3 and CCR6 expression by BJ-3105 is very less than inhibitory effect of Th differentiation. So, reduced number of T cells in CNS is mediated by less differentiation of T cells in dLN not by less expression of chemokine receptors by BJ-3105. Taken together, we concluded that BJ-3105 compound negatively controls the differentiation of CD4^+^ T cells in the EAE inductive phase and prevents EAE disease progression.

### BJ-3105 regulates CD4^+^ T cell differentiation through modulating JAK-STAT pathway

**Fig 5:**
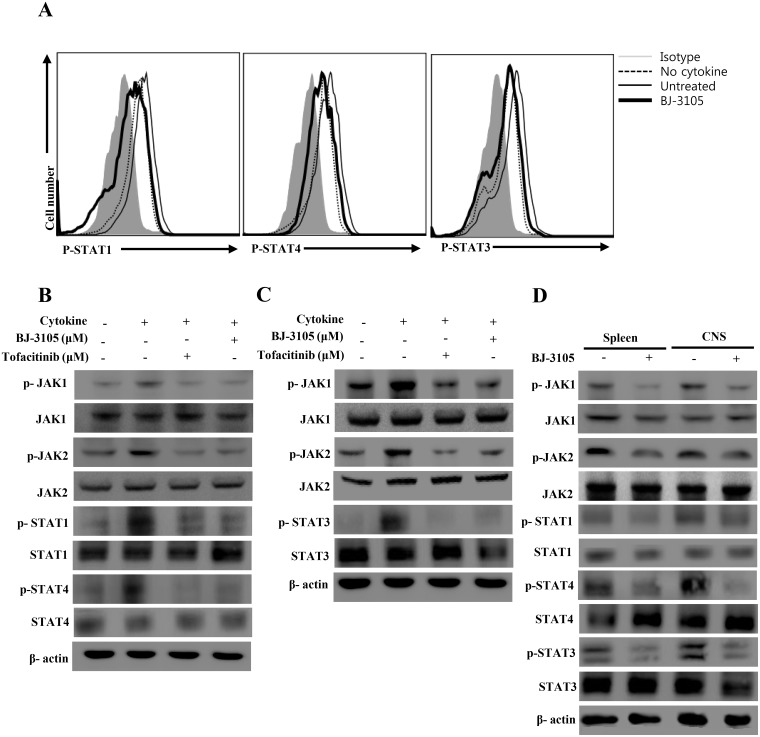
BJ-3105 controls T cell differentiation through inhibiting phosphorylation of JAK-STAT signaling pathway. Naïve CD4^+^ T cells from spleens and draining lymph nodes were isolated and cells were cultured in Th1 and Th17 cells differentiation conditions with BJ-3105. Drug and cytokine untreated groups were used as control. (A) Naïve CD4^+^ T cells were polarized to Th1 cells with anti-CD3 (2 μg/mL), anti CD28 (1 μg/mL) and IL-12 (10 ng/mL) plus anti-IL4 Ab (5 μg/mL) for 72 h of culture. Th17 cells were differentiated with anti-CD3 (2 μg/mL), anti CD28 (1 μg/mL), TGF-β (1 ng/mL), IL-6 (10 ng/mL) plus anti-IL4 Ab (5 μg/mL) and anti-IFN-γ Ab (5 μg/mL) for 72 h of culture. Phosphorylated STAT1 and STAT4 from Th1 cells and STAT3 from Th17 cells were analyzed by flow cytometer. (B) Phosphorylated and total JAK1/2, STAT1 and STAT4 were detected by immuno-blotting under Th1 polarizing condition. (C) Phosphorylated and total JAK1/2 and STAT3 were detected by immuno-blotting under Th17 polarizing condition. (D) Western blot analysis of phosphorylated and total JAK1, JAK2, STAT1, STAT3 and STAT4 proteins in splenocytes and CNS infiltrated mononuclear cells from EAE induced mice. β-actin as loading control were detected by immune-blotting. Representative results of three experiments are shown.

Consistent with the observed efficacy of BJ-3105 in EAE as a marked decrease in the percentage of Th1 and Th17 cells in EAE mice, we investigated whether this compound regulates signaling pathway in T cell differentiation. JAK-STAT signaling pathway is indispensable for controlling cytokine production and differentiation of T cells [37, 38]. We first hypothesized that BJ-3105 exerted regulatory effect on Th1 and Th17 development through direct inhibition of JAK/STAT pathway in T cells. To this end, CD4^+^ T cells were cultured with TCR stimulation and IL-12 plus BJ-3105 for three days. Autocrine IFN-γ and added IL-12 can induce the phosphorylation of STAT1 and STAT4 respectively [10]. In this condition, BJ-3105 inhibited the phosphorylation of both of STAT1 and STAT4 on day 3 after stimulation (Fig 5A). Inhibitory intensity of phosphorylation of STATs by BJ-3105 is comparable with that of JAK inhibitor, tofacitinib. Phosphorylation of STAT3 on Th17 polarizing condition was also inhibited by BJ-3105 and tofacitinib. To further explore molecular mechanisms, we analyzed the JAK which is the upstream molecule of STAT protein. As shown in the Fig 5B and 5C, consistent with the reduction in Th1 and Th17 cells, expressions of p-JAK1/2, p-STAT1 and p-STAT4 for Th1 cells and p-STAT3 for Th17 cells was decreased by BJ-3105. To further delineate the *in vivo* effect of BJ-3105, we analyzed the splenocytes and CNS mononuclear cells from BJ-3105 treated and untreated EAE mice. The result demonstrated that BJ-3105 treatment inhibit the phosphorylation of JAK1/2, p-STAT1, p-STAT3 and p-STAT4 without altering total JAK and STAT in EAE mice (Fig 5D). From the above findings, we concluded that BJ-3105 affects the JAK/STAT signaling pathway for negatively regulating the development of Th1 and Th17 cells.

## Discussion

In this study, we demonstrated that BJ-3105 had unique anti-inflammatory properties and therapeutic potential for autoimmune inflammatory conditions. We recently reported that 6-amino-2,4,5-trimethylpyridin-3-ols, first designed as antioxidants, showed good to excellent inhibitory effects against angiogenesis [39, 40]. We also found that certain 6-amino-2,4,5-trimethylpyridin-3-ols potently alleviated a rat model of 2,4,6-trinitrobenzenesulfonic acid (TNBS)-induced colitis [unpublished results]. However their roles in immune system and, more specifically, in the differentiation and function of Th cell subsets in autoimmune disease settings are still elusive. BJ-3105 has a close structural similarity to 6-amino-2,4,5-trimethylpyridin-3-ols but contains an alkoxy moiety at 6-position of pyridine ring instead. The compound was selected in a primary screening using *in vitro* Th differentiation assay along with many kinds of pyridin-3-ols. Our initial series of experiments on differentiation of the naïve CD4^+^ T cell into effector lineages utilizes specifically formulated *in vitro* system where TCR signaling and cytokine co-stimulation can drive polarization in Th1 and Th17 cell lineages. Using this approach, we found that BJ-3105 treatment significantly impaired differentiation of naïve CD4^+^ T cell into Th1, Th2 and Th17 subsets whereas the percentage of Treg cells were not affected and IL-9 production is increased by this compound. Thus, BJ-3105 has an advantage for regulating inflammatory T cells but not all T cells. Innate immune system is important for protecting host but also can be involved in inflammatory diseases. So, we evaluate the effect of BJ-3105 on innate cell activation. But BJ-3105 compound did not affect mRNA level of several cytokines of BMDC by LPS (S2 Fig). Based on our results, we believe BJ-3105 may be restricted to inflammatory T cells, Th1, Th2 and Th17.

The effector mechanism of inflammation in EAE process mostly depends on the expression of proinflammatory cytokines and chemokines. Autoimmune diseases like MS, inflammatory bowel disease and rheumatoid arthritis are the prototypic Th1 and Th17 mediated autoimmune disorders [41–43]. The EAE model recapitulates several aspects of human MS, with increased proinflammatory cytokines, particularly IFN-γ and IL-17A [35]. Although the exact role of Th1 and Th17 cells in EAE development is not well established, these effector T cells cause CNS inflammation and demyelination [6]. IFN-γ producing Th1 cells and IL-17A producing Th17 cells are very critical to autoimmune disease by inducing inflammation [44–46]. Therefore investigating specific drugs targeting Th1 and Th17 cell for management of autoimmune disease has clinical significance. We provide the *in vitro* and *in vivo* evidence that BJ-3105 represses the development of Th1 and Th17 cells and ameliorates EAE. BJ-3105 treatment significantly reduced the CD4^+^ cell population in spleen and CNS and proportion of both IFN-γ secreting Th1 and IL-17A secreting Th17 cells at the peak of disease in the spleen, draining lymph nodes, and CNS of EAE mice. The reduction of inflammatory lesion and demyelination in brain and spinal cord of BJ-3105 treated EAE mice is caused by the inhibition of Th1 and Th17 generation. These results substantially boost our understanding of the beneficial effect of BJ-3105 on management of EAE, which in turn helps to manage the effective preventive therapeutic approach to combat T cell mediated autoimmune diseases.

Encephalitogenic antigen myelin oligodendrocyte glycoprotein (MOG) has clinical relevance especially in MS [47]. Tolerance mediated treatment strategies through injection of MOG into anterior chamber of eye could be used as therapeutic tool in the treatment of MS [48, 49]. Several studies showed that autoimmune disease like multiple sclerosis can be attenuated by administration of granulocyte-macrophage-colony-stimulation factor (GM-CSF) [50]. GM-CSF acts as pro-inflammatory or regulatory cytokine that can differentiate bone-marrow precursor into tolerogenic dendritic cells which in turn can increase Treg cell number and function [51, 52]. However our compound is unique by its properties that BJ-3105 specifically inhibits Th1 and Th17 without altering Treg generation and mRNA expression of major cytokines produced by bone marrow derived dendritic cells. Therefore, compounds like BJ-3105 should receive a great deal of attention due to its excellent anti-inflammatory activities.

In cellular mechanisms, we examined the proliferation and apoptosis of T cells after BJ-3105 treatment. But BJ-3105 compound didn’t affect the activation of T cells as well as proliferation and apoptosis. In *in vitro* system, anti-CD3 Ab and anti-CD28 Ab can stimulate TCR on T cells and these T cells can be activated via CD25 expression. Tofacitinib, JAK inhibitor, suppressed the expression of CD25 after TCR stimulation but BJ-3105 didn’t (Figs 1B and 2A). Thus, tofacitinib may regulate TCR signaling but BJ-3105 do not. So, early activation and proliferation or apoptosis were not affected by BJ-3105 treatment. But *in vivo* T cell proliferation was less in BJ-3105 treated EAE mice and total cell number is also reduced in BJ-3105 treated EAE mice. *In vivo* condition is more complicated and we believe that less T cell population in BJ-3105 treated EAE mice is related with less inflammatory cytokines not by less TCR signaling or IL-2R signaling by this compound. So, based on these results, we focused on cytokine signaling.

Several studies showed that most cytokines engaged in EAE, comprising IFN-γ, IL-17A, IL-12, IL-23, use JAK/STAT signaling pathway to incite the biological response [31, 53]. Naive CD4^+^ T cells constitutively express IFN-γ receptor (IFN-γR) where IFN-γ bind and results in STAT1 phosphorylation (p-STAT1). Likewise, IL-12 binds to IL-12 receptor and results in phosphorylation of STAT4 (p-STAT4). Finally p-STAT1 and p-STAT4 bind IFN-γ promoter and drive Th1 differentiation [6]. IFN-γ-STAT1/STAT4 signaling is important in T cell mediated immune response [54]. This study demonstrated that BJ-3105 treatment inhibited IFN-γ-STAT1/STAT4 signaling pathway, consequently reducing Th1 cell differentiation. However, time point inhibition of phosphorylation in STAT1 and STAT4 was different. In time-dependent approach, BJ-3105 inhibited the phosphorylation of STAT1 at early time (12 h after stimulation) but STAT4 phosphorylation was highly suppressed at 24 h after stimulation (S4 Fig) and the inhibition of STAT3 by BJ-3105 was less at 12 h but high since then 24 h after stimulation (S4 Fig). In addition, phosphorylation of JAK was also affected by BJ-3105. So this compound regulate JAK/STAT signaling pathway. However, still it is unclear whether inhibition of phosphorylation is a direct or indirect effect of the drug on JAK and STAT molecules. Compared with tofacitinib, JAK inhibitor, the effect of BJ-3105 is different with tofacitinib on T cell activation. Therefore, BJ-3105 may have an unknown target for regulating JAK/STAT signaling.

BJ-3105 not only regulates the priming of pathogenic Th1 and Th17 in early stage of EAE but also suppresses these subsets at the peak of disease through inhibition of JAK/STAT pathways. BJ-3105, a novel anti-inflammatory agent, inhibits Th1 and Th17 differentiation *in vitro*, prevents onset, and ameliorates disease progression in EAE inflicted mice through sustained inhibition of JAK1, JAK2, STAT1, STAT4 and STAT3 phosphorylation. Our findings suggest that BJ-3105 is a promising therapeutic compound for the management of Th1 and Th17 mediated inflammation and autoimmune disease.

## Supporting Information

S1 Fig BJ-3105 potently inhibits Th2 but not Th9 cell Differentiation. Purified naïve mouse CD4^+^ T cells from spleens and draining lymph nodes were stimulated in Th2 and Th9 polarizing conditions for 72 h in the presence of BJ-3105 (1 μM) or tofacitinib (1 μM). CD4^+^ T cells were then restimulated with PMA, ionomycin in the absence of golgistop for 24 h and analyzed by flow cytometer. The untreated controls were cultured in the presence of DMSO. IL-4, IL-5 and IL-13 from Th2 and IL-9 from Th9 polarizing cells were quantified by cytokine binding assay. Plots are Mean±SEM of triplicate samples. Data are representative of two independent experiments. *p < 0.05, **p < 0.01, compared with drug untreated group.
(TIF)

S2 Fig Effect of BJ-3105 on the mRNA expression of various cytokines upon LPS stimulation. Bone marrow-derived dendritic cells were stimulated with 200 ng/ml of LPS in the presence of vehicle or BJ-3105 (10 μM) for 4 h to examine mRNA expression. The mRNA levels of the indicated genes were analyzed by quantitative RT-PCR. Representative results of three experiments are shown.
(TIF)

S3 Fig BJ-3105 decrease T cell infiltration and demyelination in CNS and decrease CD4^+^ T cells proliferation *in vivo*. (A) Brain and spinal cord section obtained from the normal control mice, EAE mice or BJ-3105 treated EAE mice at day 15 postimmunization were analyzed by H&E staining for inflammation and Luxol Fast Blue staining for demyelination. Data presented are representative of three independent experiment. (B) BrdU was incorporated *in vivo* through drinking water in EAE mice and BrdU^+^CD4^+^ T cells were shown in spleen and CNS. *p < 0.05, compared with drug untreated group. Representative results of three experiments are shown.
(TIF)

S4 Fig BJ-3105 reduces Naïve CD4^+^ T cell differentiation by inhibiting the phosphorylation STAT. Naïve CD4^+^ T cells from spleens and draining lymph nodes were isolated and cells were cultured in Th1 and Th17 cells differentiation conditions with BJ-3105. Drug and cytokine untreated groups were used as control. (A) Phosphorylated and total STAT1 and STAT4 were detected by immuno-blotting under Th1 polarizing condition in 12 h, 24 h and 48 h of culture with different dose of BJ-3105. (B) p-STAT3 and total STAT3 were detected under Th17 polarizing condition in 12 h, 24 h and 48 h of culture. β-actin as loading control were detected by immune-blotting. Representative results of three experiments are shown.
(TIF)
