# Supplementary figures and images for: BJ-3105, a 6-Alkoxypyridin-3-ol Analog, Impairs T Cell Differentiation and Prevents Experimental Autoimmune Encephalomyelitis Disease Progression
Source: PLoS One. 2017 Jan 17;12(1):e0168942. doi: 10.1371/journal.pone.0168942 (PMC5241145; doi:10.1371/journal.pone.0168942)

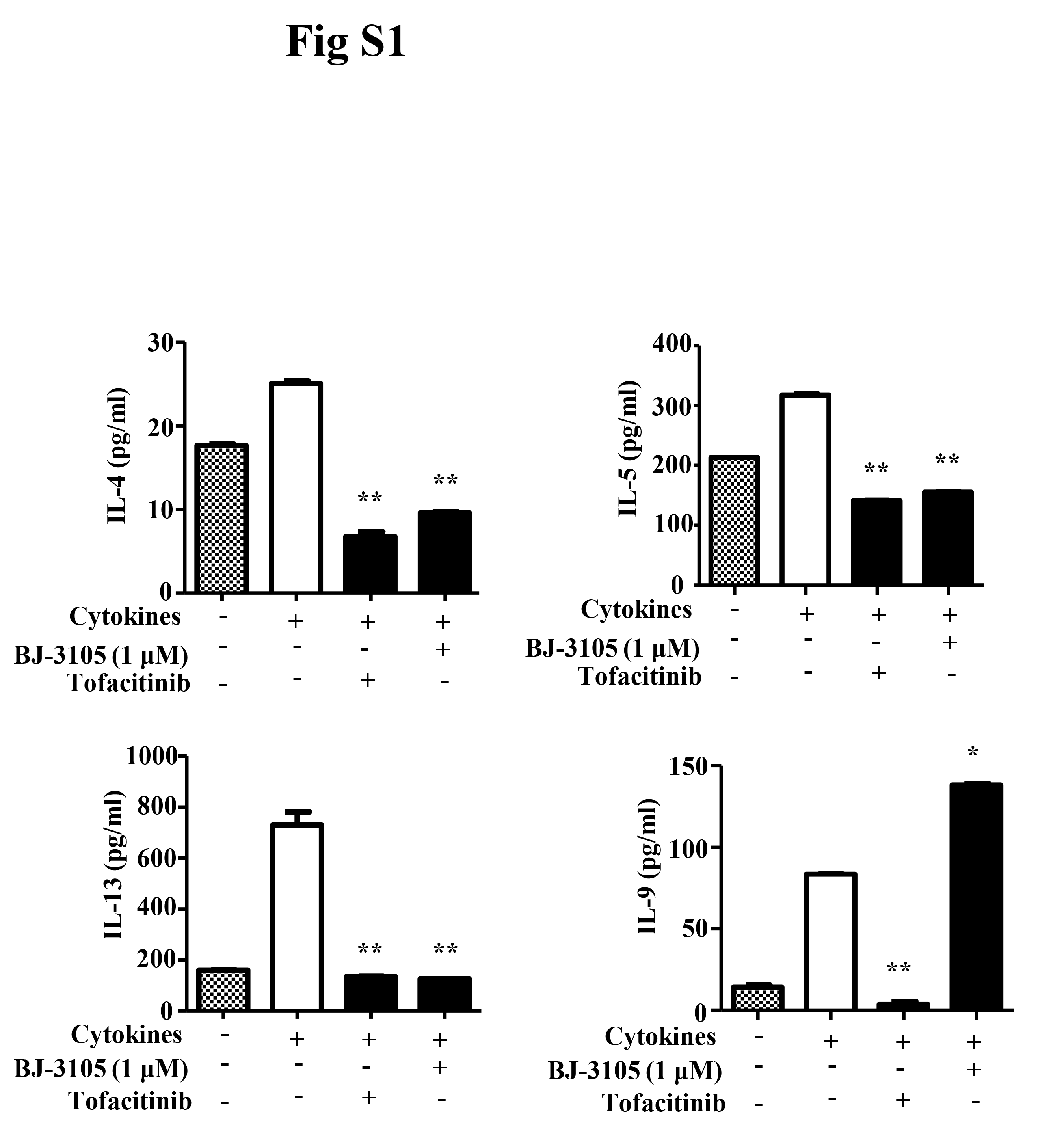

Supplement: S1 Fig — Purified naïve mouse CD4+ T cells from spleens and draining lymph nodes were stimulated in Th2 and Th9 polarizing conditions for 72 h in the presence of BJ-3105 (1 μM) or tofacitinib (1 μM). CD4+ T cells were then restimulated with PMA, ionomycin in the absence of golgistop for 24 h and analyzed by flow cytometer. The untreated controls were cultured in the presence of DMSO. IL-4, IL-5 and IL-13 from Th2 and IL-9 from Th9 polarizing cells were quantified by cytokine binding assay. Plots are Mean±SEM of triplicate samples. Data are representative of two independent experiments. *p < 0.05, **p < 0.01, compared with drug untreated group. (TIF) [file pone.0168942.s001.tif]

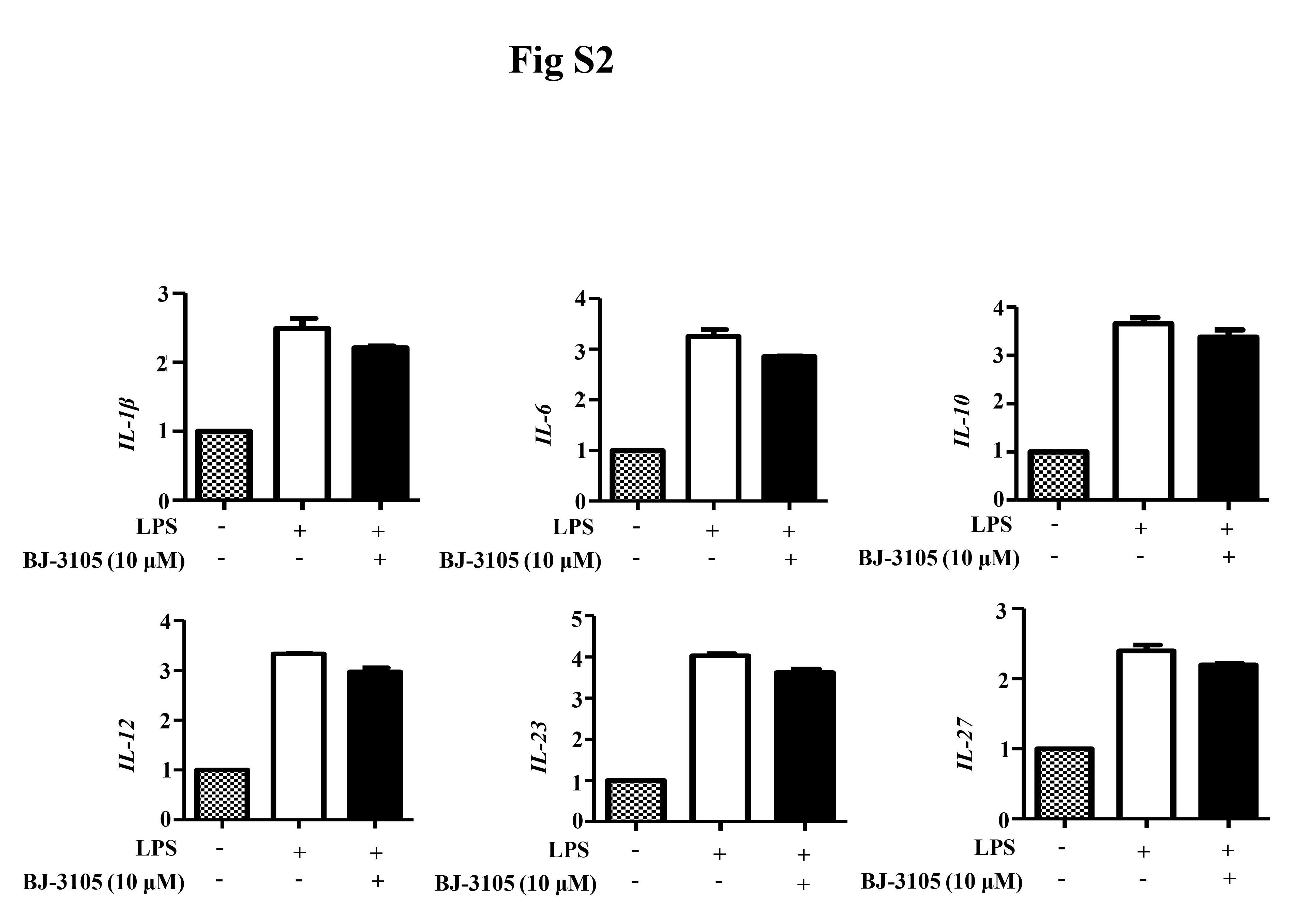

Supplement: S2 Fig — Bone marrow-derived dendritic cells were stimulated with 200 ng/ml of LPS in the presence of vehicle or BJ-3105 (10 μM) for 4 h to examine mRNA expression. The mRNA levels of the indicated genes were analyzed by quantitative RT-PCR. Representative results of three experiments are shown. (TIF) [file pone.0168942.s002.tif]

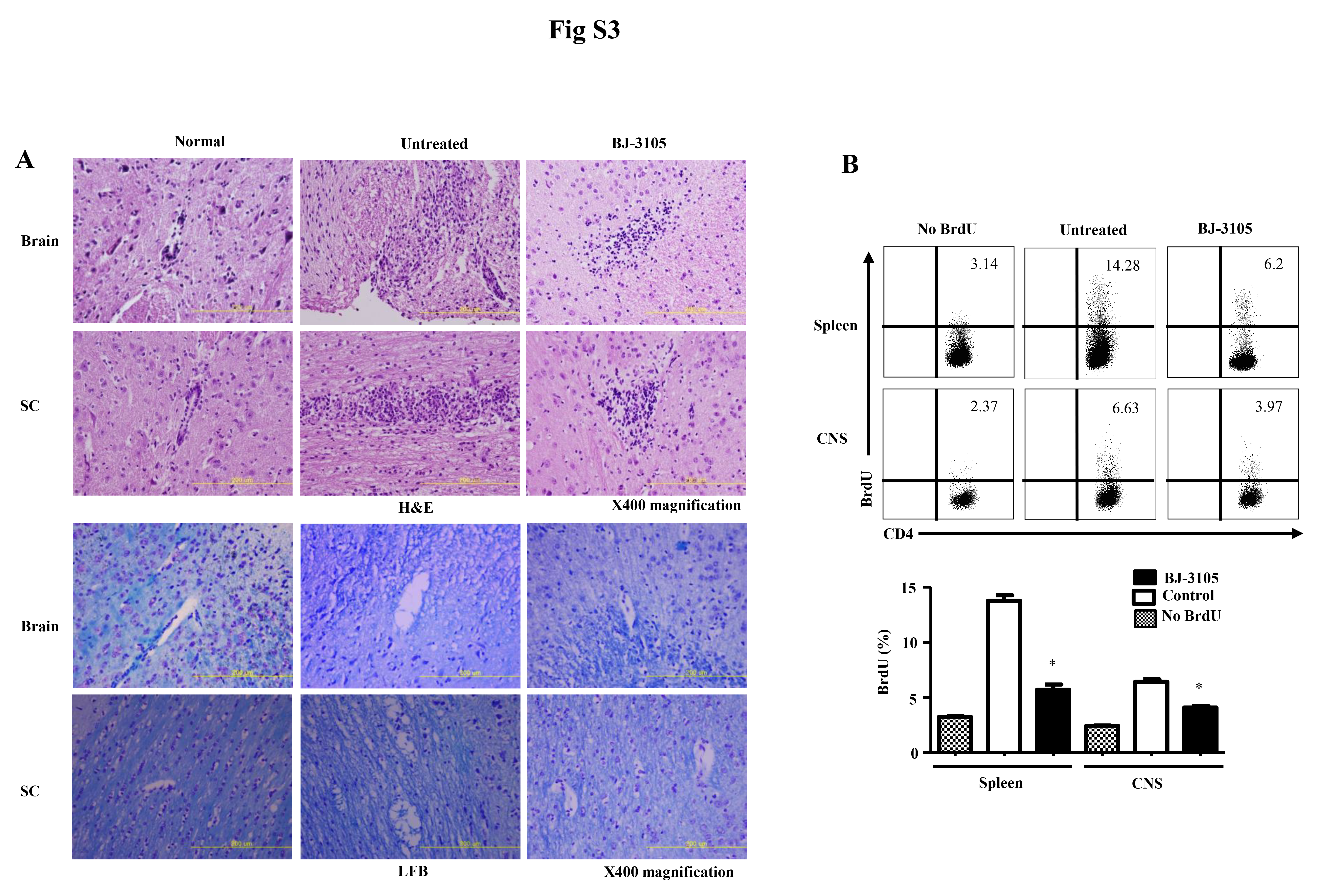

Supplement: S3 Fig — (A) Brain and spinal cord section obtained from the normal control mice, EAE mice or BJ-3105 treated EAE mice at day 15 postimmunization were analyzed by H&E staining for inflammation and Luxol Fast Blue staining for demyelination. Data presented are representative of three independent experiment. (B) BrdU was incorporated in vivo through drinking water in EAE mice and BrdU+CD4+ T cells were shown in spleen and CNS. *p < 0.05, compared with drug untreated group. Representative results of three experiments are shown. (TIF) [file pone.0168942.s003.tif]

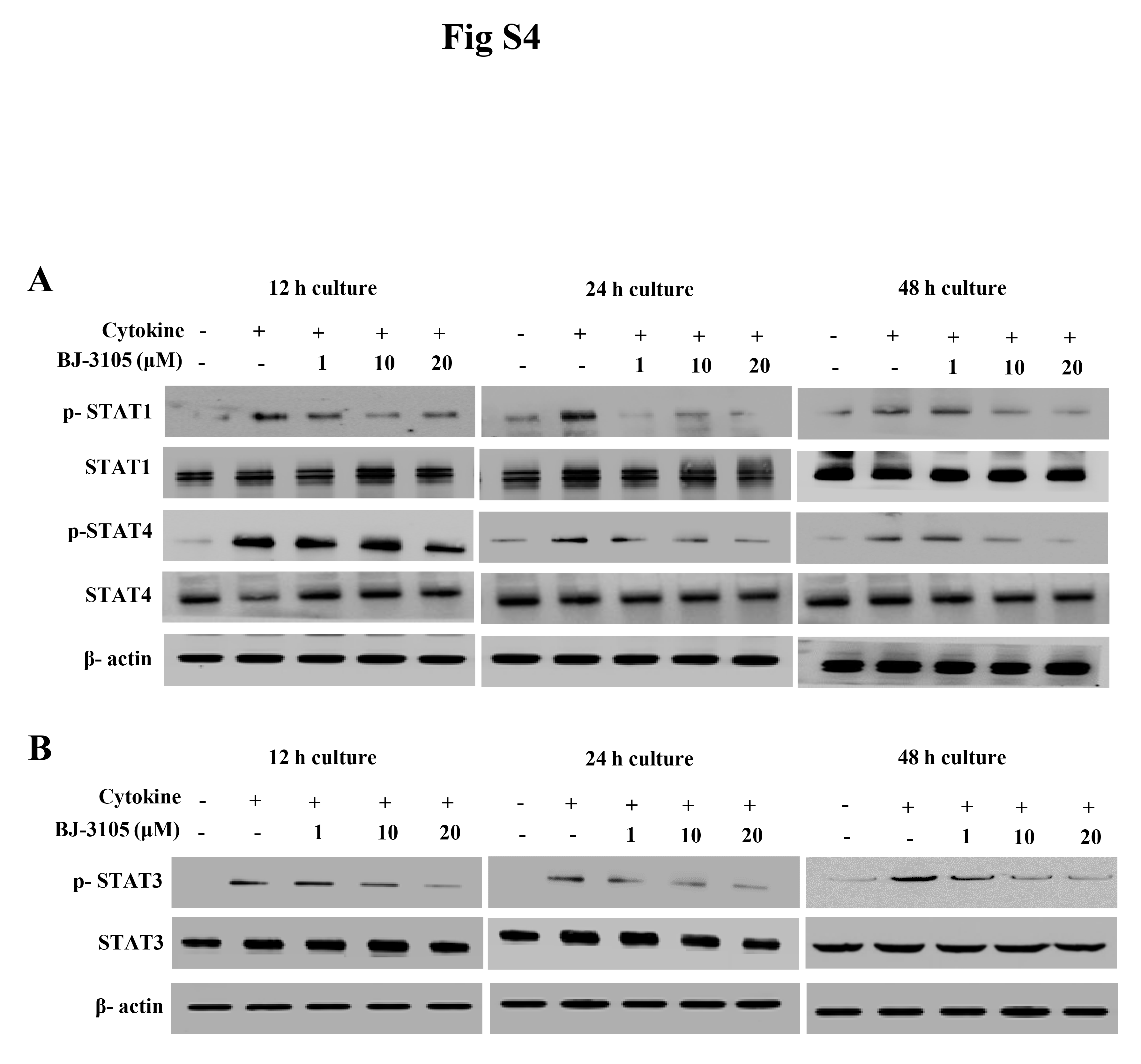

Supplement: S4 Fig — Naïve CD4+ T cells from spleens and draining lymph nodes were isolated and cells were cultured in Th1 and Th17 cells differentiation conditions with BJ-3105. Drug and cytokine untreated groups were used as control. (A) Phosphorylated and total STAT1 and STAT4 were detected by immuno-blotting under Th1 polarizing condition in 12 h, 24 h and 48 h of culture with different dose of BJ-3105. (B) p-STAT3 and total STAT3 were detected under Th17 polarizing condition in 12 h, 24 h and 48 h of culture. β-actin as loading control were detected by immune-blotting. Representative results of three experiments are shown. (TIF) [file pone.0168942.s004.tif]
